# ^1^H NMR-based metabolomics reveals the effect of maternal habitual dietary patterns on human amniotic fluid profile

**DOI:** 10.1038/s41598-018-22230-y

**Published:** 2018-03-06

**Authors:** Maria Fotiou, Charalambos Fotakis, Foteini Tsakoumaki, Elpiniki Athanasiadou, Charikleia Kyrkou, Aristea Dimitropoulou, Thalia Tsiaka, Anastasia Chrysovalantou Chatziioannou, Kosmas Sarafidis, George Menexes, Georgios Theodoridis, Costas G. Biliaderis, Panagiotis Zoumpoulakis, Apostolos P. Athanasiadis, Alexandra-Maria Michaelidou

**Affiliations:** 1 Department of Food Science and Technology, School of Agriculture, Aristotle University of Thessaloniki, Thessaloniki, Greece; 2 Institute of Biology, Medicinal Chemistry and Biotechnology, National Hellenic Research Foundation, Athens, Greece; 3 1st Department of Obstetrics and Gynecology, School of Medicine, Aristotle University of Thessaloniki, Thessaloniki, Greece; 4 School of Chemistry, Aristotle University of Thessaloniki, Thessaloniki, Greece; 5 1st Department of Neonatology, School of Medicine, Aristotle University of Thessaloniki, Thessaloniki, Greece; 6 Department of Field Crops and Ecology, School of Agriculture, Aristotle University of Thessaloniki, Thessaloniki, Greece; 7 3rd Department of Obstetrics and Gynecology, School of Medicine, Aristotle University of Thessaloniki, Thessaloniki, Greece

## Abstract

Maternal diet may influence offspring’s health, even within well-nourished populations. Amniotic fluid (AF) provides a rational compartment for studies on fetal metabolism. Evidence in animal models indicates that maternal diet affects AF metabolic profile; however, data from human studies are scarce. Therefore, we have explored whether AF content may be influenced by maternal diet, using a validated food-frequency questionnaire and implementing NMR-based metabolomics. Sixty-five AF specimens, from women undergoing second-trimester amniocentesis for prenatal diagnosis, were analysed. Complementary, maternal serum and urine samples were profiled. Hierarchical cluster analysis identified 2 dietary patterns, cluster 1 (C1, n = 33) and cluster 2 (C2, n = 32). C1 was characterized by significantly higher percentages of energy derived from refined cereals, yellow cheese, red meat, poultry, and “ready-to-eat” foods, while C2 by higher (P < 0.05) whole cereals, vegetables, fruits, legumes, and nuts. ^1^H NMR spectra allowed the identification of metabolites associated with these dietary patterns; glucose, alanine, tyrosine, valine, citrate, *cis*-acotinate, and formate were the key discriminatory metabolites elevated in C1 AF specimens. This is the first evidence to suggest that the composition of AF is influenced by maternal habitual dietary patterns. Our results highlight the need to broaden the knowledge on the importance of maternal nutrition during pregnancy.

## Introduction

Over the past decades, the field of nutritional epidemiology has generated a large body of evidence indicating that maternal nutrition plays a critical role in fetal growth^1–4^ and pregnancy outcome^5^. Research regarding the phenomenon termed “fetal programming” and the theory of “fetal origins of adult disease” has initially focused on the impact of maternal overnutrition or undernutrition on the growth potential in utero^1–4^. However, emerging data^6–9^ indicate that even small alterations in dietary quality or quantity may be associated with significant shifts in the fetal environment, probably related to increased vulnerability to chronic diseases in adult life.

Amniotic fluid (AF) provides a rational compartment for studies on fetal nutrition and metabolism, since its composition reflects both maternal health and fetal status^10–13^. Scientific evidence^10^ indicates that this biofluid is a complex and dynamic milieu containing nutrients essential for fetal growth; its composition is changing, as pregnancy progresses, and, at the second half of gestation, its content and volume are affected by several factors including fetal urination and swallowing, as well as, fetal skin keratinisation^10^.

The potential effect of maternal diet on the nutrient composition of AF has been demonstrated in animal studies^14–19^. In particular, pregnant rats exhibited a significant increase in AF glucose and decrease in uric acid, as the level of carbohydrate increased in the maternal diet^14^. On the contrary, maternal dietary glucose restriction in rats resulted in the reduction of AF methionine and phenylalanine^15^. Similarly, maternal nutrient restriction in ewes markedly reduced total amino acids and polyamines concentration in AF^16^, while, a “famine diet” in rats also influenced AF composition^17^. Furthermore, Friesen and Innis^18^ demonstrated that maternal fat intake alters AF and fetal intestinal membrane essential n-6 and n-3 fatty acids in rats. In addition, in a very recent study in sows^19^, chitosan oligosaccharide supplementation induced AF metabolic profile modifications.

To the best of our knowledge, published data on the effect of maternal nutrition on human AF composition are only limited to the study by Felig *et al*.^20^, reporting changes in AF, after 84–90 hours of fasting. Nevertheless, it should be highlighted that the effect of maternal habitual diet on the composition of human AF has not been yet explored.

Metabolomics is a bio-analytical approach that allows the identification of a large number of metabolites in biological matrices, essentially reflecting biological processes of the organism^21–23^. In prenatal medicine, metabolomics of human blood, urine, and AF have been used for the evaluation/prognostication of fetal malformations, preterm delivery, and other pregnancy complications^12,24–40^. Furthermore, within the same research field, Wan *et al*.^19^ reported that AF metabolomics provides novel insights into the diet-regulated fetal survival and growth in a pig model study.

Hence, the challenge for us was to explore whether maternal habitual dietary patterns influence the composition of human AF. To accomplish this task, we used a validated food-frequency questionnaire^41^ and applied ^1^H NMR-based metabolomics. It is of interest to note that, although amniocentesis is an invasive procedure employed only under specific indications, the realization that AF content may be influenced by maternal diet would advance the knowledge on the importance of maternal nutrition during pregnancy.

## Results

### Identification of dietary clusters

**Figure 1:**
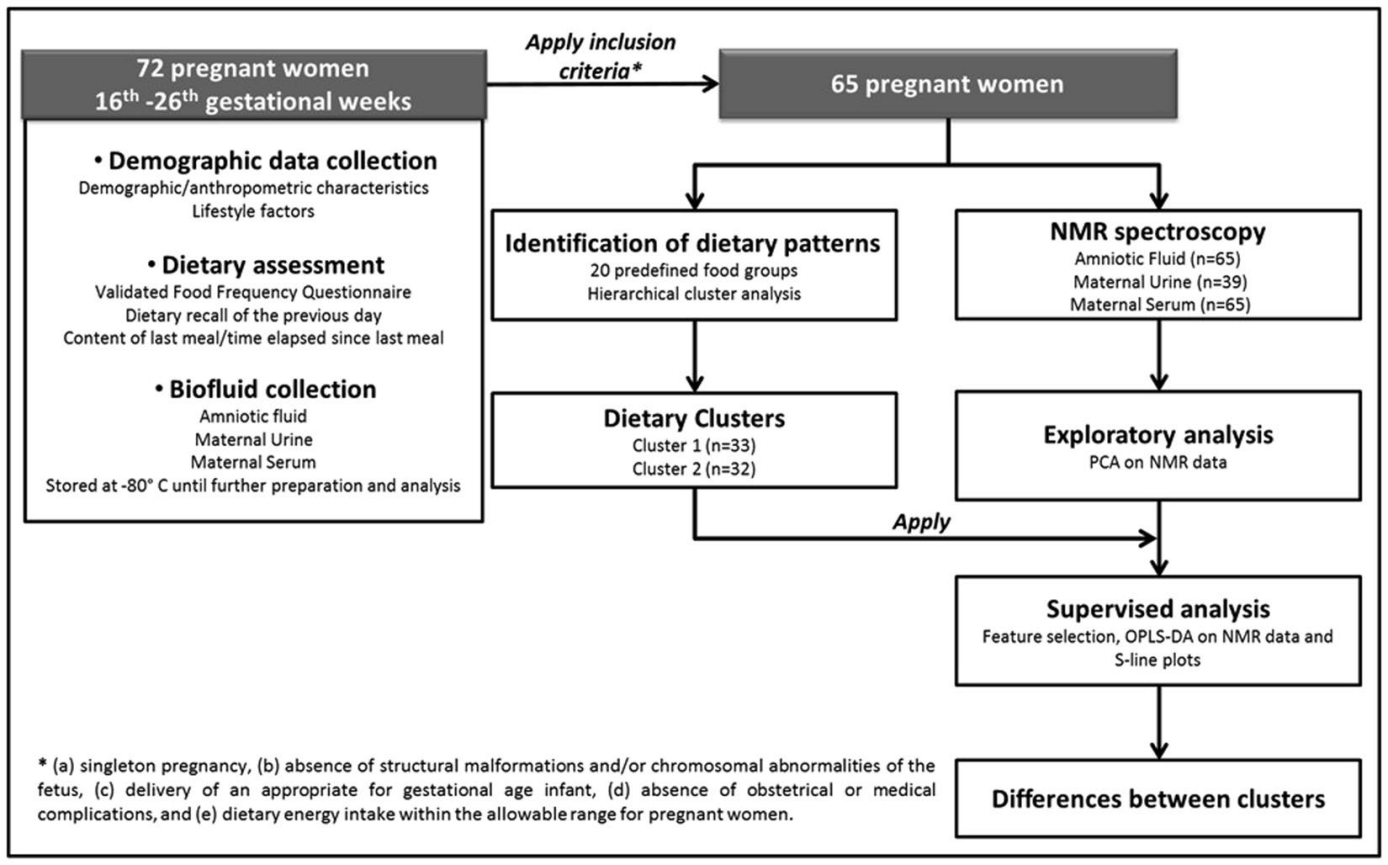
Flow diagram of the study.

Sixty-five women were included in the present study, as shown in the flow diagram (Fig. 1). Two interpretable and statistically significant (upper tail rule: *t* = 39.85, d*f* = 63, P < 0.001) dietary patterns were identified through Hierarchical Cluster Analysis (HCA). Thirty-three women were grouped in cluster 1 (C1), while 32 in cluster 2 (C2). The Discriminant Analysis indicated good classification ability of the selected cluster solution, since the agreement between actual and predicted cluster allocation was 93.8%. C1 and C2 differed (P < 0.05) in the percentages of energy contributed by 10 out of the 20 predefined food groups (Table 1). C1 had higher intakes of refined cereals, yellow cheese, red meat, poultry, and “ready-to-eat” foods (P < 0.05). The macro- and micro- nutrient intakes, as well as selected dietary indices, reflecting these dietary preferences, are given in Table 2. As indicated, C1 had significantly higher energy contributions from total protein, animal protein, and saturated fatty acids. Additionally, the intake of heme iron was elevated compared to that of C2 (P < 0.05). Dietary glycaemic index (GI) was also higher in C1 (P < 0.05). Instead, C2 was characterized by significantly higher percentages of energy derived from plant protein, monounsaturated and polyunsaturated fatty acids (P < 0.05) (Table 2). These differences in energy generating nutrients, in combination with the higher intake of fibre, folate, vitamin C, vitamin E, magnesium, potassium, and non heme iron (P < 0.05) in C2 (Table 2) may ensue from the significantly higher energy contributions from whole cereals, vegetables, fruits, legumes, and nuts (Table 1).

**Table 1:** Percentages of energy contribution of food groups between the two dietary clusters, cluster 1 (C1) and cluster 2 (C2). ^*^P-value < 0.05 represents significant differences in mean values according to the results of *t*-test or Mann-Whitney test indicated by †. SD: standard deviation.

Food group	Energy contribution		
	C1 (n = 33) Mean ± SD	C2 (n = 32) Mean ± SD	P-value^*^
Refined cereals	13 ± 6	3.9 ± 4.0	<0.001
Whole cereals	2.1 ± 3.3	6.6 ± 4.8	<0.001†
Pasta	6.7 ± 2.8	5.5 ± 2.5	0.082
Rice	4.5 ± 2.4	4.2 ± 2.1	0.595
Vegetables	11 ± 4	16 ± 4	<0.001
Fruits	6.3 ± 3.8	9.7 ± 4.6	0.002
Dairy (milk/yogurt) low fat	2.5 ± 3.3	4.0 ± 4.8	0.296†
Dairy (milk/yogurt) full fat	4.9 ± 6.0	2.6 ± 3.8	0.129†
Feta cheese	5.1 ± 3.3	6.0 ± 3.9	0.305
Yellow cheese	3.3 ± 2.5	2.1 ± 2.3	0.049
Red meat	8.4 ± 3.9	6.6 ± 3.0	0.019
Poultry	3.2 ± 1.8	2.2 ± 1.2	0.011
High fat processed meat	0.5 ± 1.2	0.2 ± 0.4	0.117†
Low fat processed meat	0.4 ± 0.6	0.4 ± 0.7	0.501†
Eggs	0.9 ± 1.1	0.9 ± 0.9	0.999†
Fish	2.1 ± 1.5	2.2 ± 1.6	0.715
Legumes	2.7 ± 2.0	4.1 ± 2.7	0.029
Nuts	0.7 ± 1.4	4.2 ± 3.9	<0.001†
Sweets	8.4 ± 5.0	7.5 ± 5.1	0.467
“Ready-to-eat” foods	2.2 ± 1.9	1.3 ± 1.1	0.035

**Table 2:** Nutritional profile (energy, macro- and micro- nutrient intakes, dietary indices) of the two dietary clusters, cluster 1 (C1) and cluster 2 (C2). *P-value < 0.05 represents significant differences in mean values according to the results of *t*-test. SD: standard deviation; SFA: Saturated fatty acids; MUFA: Monounsaturated fatty acids; PUFA: Polyunsaturated fatty acids.

	C1 (n = 33) Mean ± SD	C2 (n = 32) Mean ± SD	P-value^*^
Energy (kcal)	1775 ± 289	1810 ± 283	0.622
% Energy from total protein	16 ± 2	14 ± 2	0.001
% Energy from plant protein	4.8 ± 0.6	5.2 ± 0.8	0.011
% Energy from animal protein	11 ± 2	8.9 ± 1.6	<0.001
% Energy from total lipids	45 ± 4	46 ± 4	0.058
% Energy from MUFA	22 ± 2	24 ± 2	0.003
% Energy from PUFA	5.6 ± 0.8	6.7 ± 1.5	<0.001
% Energy from SFA	14 ± 2	13 ± 2	0.015
% Energy from carbohydrates	38 ± 4	37 ± 4	0.516
Fibre (g)	14 ± 4	20 ± 4	<0.001
Cholesterol (mg)	240 ± 78	210 ± 58	0.086
Folate (mcg)	172 ± 50	235 ± 47	<0.001
Vitamin C (mg)	84 ± 42	124 ± 60	0.002
Vitamin E (mg)	12 ± 5	17 ± 6	<0.001
Calcium (mg)	909 ± 222	887 ± 300	0.732
Magnesium (mg)	206 ± 40	259 ± 63	<0.001
Potassium (mg)	2068 ± 446	2448 ± 532	0.003
Sodium (mg)	2073 ± 380	1886 ± 489	0.089
Iron heme (mg)	1.4 ± 0.4	1.2 ± 0.4	0.025
Iron non heme (mg)	6.2 ± 1.7	7.1 ± 1.5	0.028
Dietary Glycaemic Index	76 ± 6	71 ± 7	0.004
Dietary Glycaemic Load	128 ± 35	119 ± 24	0.216
Time since last meal (hour)	2.9 ± 1.2	3.1 ± 1.2	0.520

The demographic/anthropometric and clinical characteristics of the two dietary clusters are presented in Table 3; a borderline statistically significant difference was recorded for ponderal index (P = 0.076).

**Table 3:** Demographic/anthropometric and clinical characteristics of the 65 participants and their offspring for the two dietary clusters, cluster 1 (C1) and cluster 2 (C2). *P-value < 0.05 represents significant differences in mean values according to the results of *t*-test. SD: standard deviation; BMI: body mass index.

Characteristics	C1 (n = 33) Mean ± SD	C2 (n = 32) Mean ± SD	P-value^*^
Maternal age (year)	36 ± 5	37 ± 4	0.332
Pre-pregnancy BMI (kg/m^2^)	25.8 ± 6.6	24.2 ± 4.9	0.274
Women weight change until amniocentesis (kg)	3.8 ± 4.4	4.8 ± 2.5	0.265
Amniocentesis age (week)	19 ± 2	19 ± 1	0.751
Estimated fetal weight (g)	300 ± 100	301 ± 99	0.996
Gestational age at birth (week)	38 ± 1	39 ± 2	0.769
Birthweight (g)	3129 ± 424	3144 ± 642	0.914
Neonatal length (cm)	50.2 ± 2.1	49.7 ± 2.2	0.385
Birth weight centile	42 ± 23	41 ± 27	0.946
Ponderal index (100*g/cm^3^)	2.5 ± 0.2	2.6 ± 0.3	0.076

### Analysis of ^1^H NMR Spectroscopic Data

**Figure 2:**
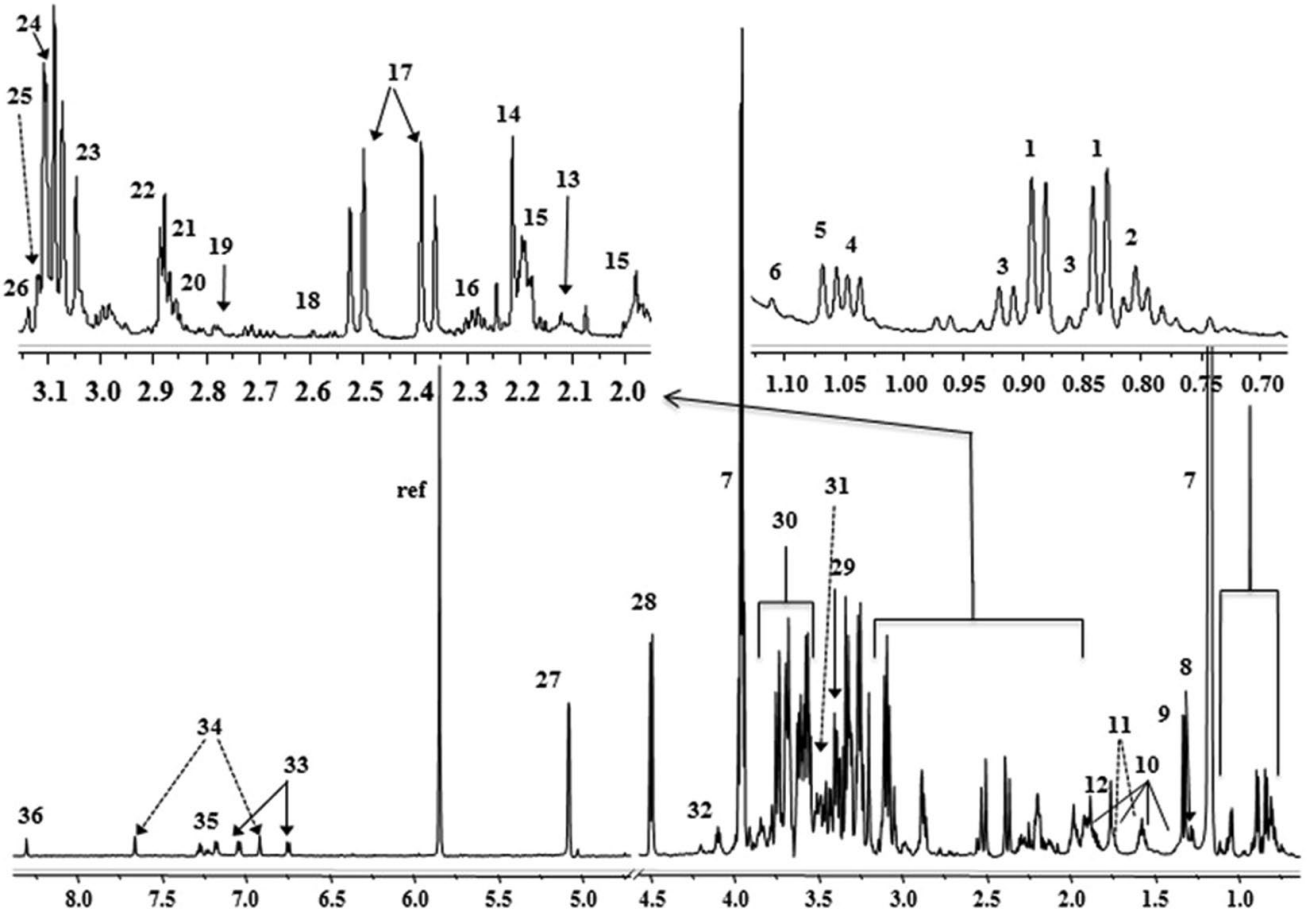
^1^H NMR spectra of amniotic fluid sample with annotation on the identified metabolites. 1:valine; 2:leucine; 3:isoleucine; 4:isobutyrate; 5:2-hydroxy-3-methylbutyrate; 6:2-hydroxybutyrate; 7:lactate; 8:3-hydroxybutyrate; 9:alanine; 10:lysine; 11:arginine; 12:acetate; 13:acetone; 14:acetoacetate; 15:glutamine; 16:glutamate; 17:citrate; 18:methylamine; 19:aspartate; 20:dimethylamine; 21:creatine; 22:creatinine; 23:choline; 24:phosphocholine; 25:betaine; 26:methanol; 27:α-D-glucose; 28:β-D-glucose; 29:glycine; 30:glycerol; 31:myo-inositol; 32:threonine; 33:tyrosine; 34:histidine; 35:phenylalanine; 36:formate.

**Figure 3:**
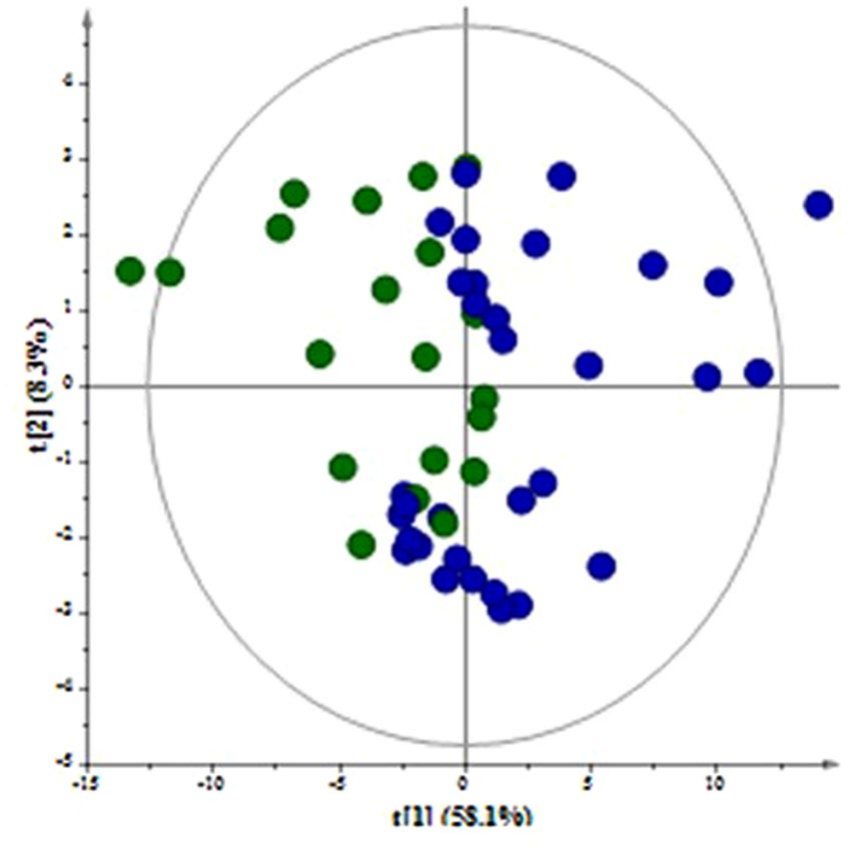
PCA model of amniotic fluid samples. A = 5; N = 58; R^2^(cum) = 0.82; Q^2^(cum) = 0.70.  Cluster 1 (C1)  Cluster 2 (C2).

Typical standard ^1^H NMR spectra of human AF, urine, and serum with annotations on the identified metabolites are depicted in Fig. 2, Supplementary Figs S1 and S2, respectively. Principal Component Analysis (PCA) was implemented to provide an overview on the samples’ clustering (Fig. 3 for AF, Supplementary Fig. S3 for urine and serum). Interestingly, a clear trend for clustering of the samples was observed along the first component, which explained 58.1% of the metabolic variance in AF, 60% in urine, and 48% in serum. This clustering indicates that these unsupervised models highlighted metabolic differences in relation to the dietary patterns.

Then, we embedded the class information from the dietary clusters into Orthogonal Partial Least Squared-Discriminant Analysis (OPLS-DA) models in order to pinpoint the metabolites responsible for the discriminations. The extracted OPLS-DA models classified correctly 89% of the AF samples, 79% of the urine samples, and 83% of the serum samples.

**Figure 4:**
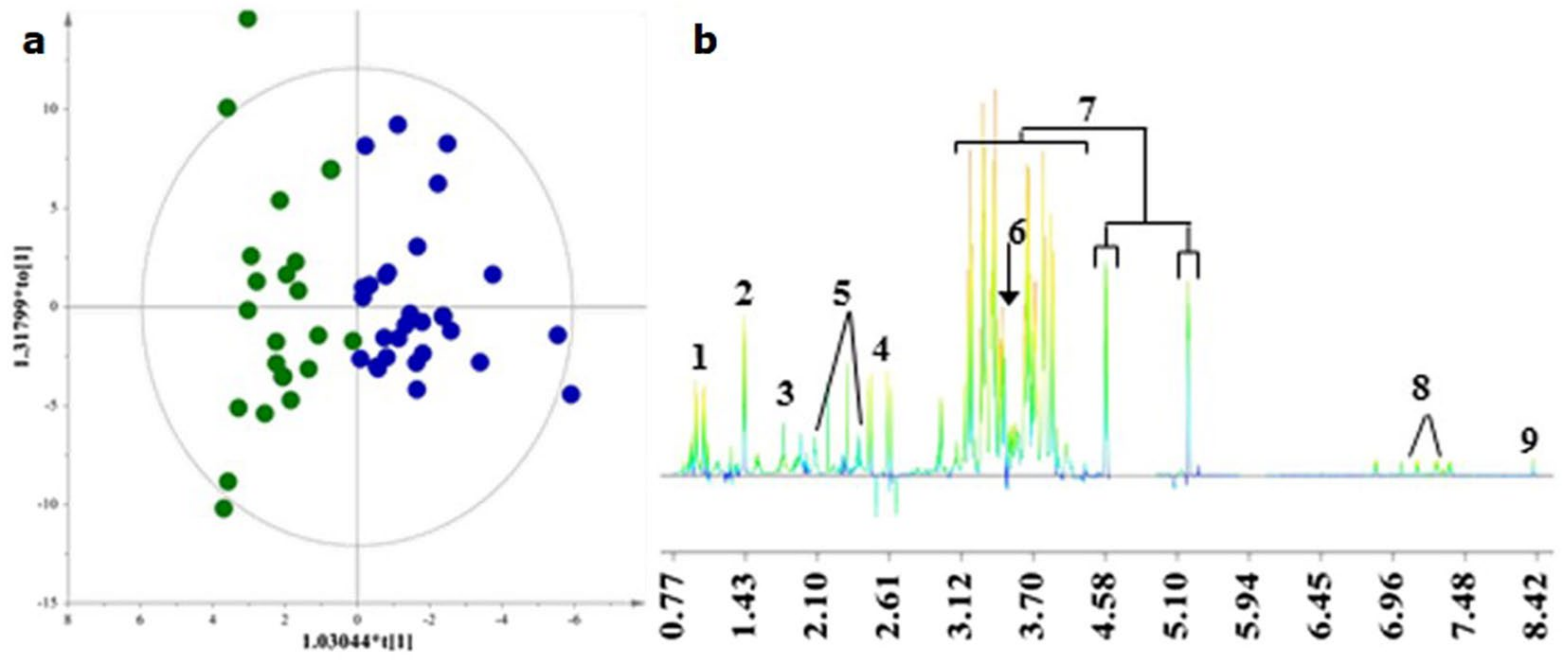
(**a**) OPLS-DA model of amniotic fluid samples. A = 1 + 1; N = 54; R^2^X(cum) = 0.66; R^2^Y(cum) = 0.76; Q^2^(cum) = 0.64.  Cluster 1 (C1)  Cluster 2 (C2). (**b**) S-Line plot (1:valine; 2:alanine; 3:acetate; 4:citrate; 5:glutamine; 6:*cis*-acotinate; 7:glucose; 8:tyrosine; 9:formate, higher in C1).

For AF, the discrimination between the two clusters was evident along the first component (Fig. 4a) and the key metabolites, which exhibited a strong correlation with C1 as depicted in the S-line plot, are presented in Fig. 4b. We extracted Receiver Operating Characteristic (ROC) curves for each metabolite, in order to elucidate the markers that express the impact of habitual diet between the two clusters and avoid false selection. In fact, glucose, alanine, tyrosine, valine, citrate, *cis*-acotinate, and formate exhibited Area Under the curve of the ROC (AUROC) > 0.7 (Table 4). These should be considered as the most fitting markers of habitual diet in the AF samples and their trends framed in box plots are presented in the Supplementary Fig. S4. Metabolites exhibiting an 0.5 < AUROC < 0.7 and AUROC < 0.5 are, also, presented in Table 4.

**Table 4:** List of metabolite changes in amniotic fluid, maternal urine, and maternal serum corresponding to the two dietary clusters, cluster 1 (C1) and cluster 2 (C2). δ (^1^H shift) ppm corresponds to signals used for integration; s: singlet; d: doublet; t: triplet; dd: doublet of doublets; m: multiplet; AUROC: Area under the curve of the receiver operating characteristic.

Substrate	Metabolites	δ (^1^H shift) ppm	Assignment	Multi-plicity	AUROC	Higher in cluster
Amniotic fluid	glucose	5.18, 4.55, 3.4–4.0	various, H1	d, d, m	>0.7	C1
	alanine	1.42	CH_3_	d	>0.7	C1
	glutamine	2.44	half γ-CH_2_	m	<0.5	C1
	tyrosine	6.8, 7.13	CH, CH	d, d	>0.7	C1
	valine	0.94, 1.03	CH_3_, CH_3_	d, d	>0.7	C1
	acetate	1.87	CH_3_	s	<0.5	C1
	citrate	2.53, 2.63	half CH_2_, half CH_2_	d, d	>0.7	C1
	*cis*-acotinate	3.45	CH_2_	d	>0.7	C1
	formate	8.4	CH	s	>0.7	C1
	histidine	7.03, 7.84	H_4_, H_2_	s, s	0.5–0.7	C1
	mannose	5.18	H_6_	d	0.5–0.7	C1
	phenylalanine	7.22, 7.33	H_2_ + H_6_, H_3_ + H_5_	m, m	0.5–0.7	C1
	1-methyl-histidine	7.07, 7.80	4-CH, 2-CH	s, s	0.5–0.7	C1
	fumarate	6.81	CH_3_	s	0.5–0.7	C1
	hippurate	7.59, 7.87	H_3_/H_5_, H_2_/H_6_	t, d	0.5–0.7	C1
Maternal urine	alanine	1.42	CH_3_	d	>0.7	C1
	glutamine	2.44	half γ-CH_2_	m	0.5–0.7	C1
	isoleucine	0.95, 1.04	δ-CH_3_, β-CH_3_	t, d	0.5–0.7	C1
	leucine	0.95	δ-CH_3_	d	0.5–0.7	C1
	lysine	1.66–1.88	δ-CH_2_, β-CH_2_	m, m	0.5–0.7	C1
	valine	0.94, 1.03	CH_3_, CH_3_	d, d	>0.7	C1
	citrate	2.53, 2.63	half CH_2_, half CH_2_	d, d	>0.7	C1
	formate	8.4	CH	s	0.5–0.7	C1
	isobutyrate	1.05, 2.41	CH_3_, CH	d, dd	>0.7	C1
	methyl-succinate	1.08	CH_3_	d	<0.5	C1
	pyroglutamate	2.39	CH_2_	m	<0.5	C1
	2-hydroxyisobutyrate	1.36	CH_3_	s	0.5–0.7	C1
	2-hydroxyglutarate	2.42	CH_2_	t	0.5–0.7	C1
	3-hydroxyisovalerate	1.26	CH_3_	s	0.5–0.7	C1
	betaine	3.21	CH_3_	s	>0.7	C1
	choline	3.14	N(CH_3_)_3_	s	>0.7	C1
	dimethylglycine	2.93	CH_3_	s	>0.7	C1
	creatine	2.98	CH_3_	s	>0.7	C1
	creatinine	3	CH_3_	s	>0.7	C1
	trimethylamine N-oxide	3.19	CH_3_	s	>0.7	C1
	urea	5.78	NH_2_ + NH_2_	m	0.5–0.7	C1
	bile acids	0.6–0.7	—	m	<0.5	C1
	imidazole	7.27	CH	s	<0.5	C1
Maternal serum	cholesterol-VLDL	0.7	C_18_-CH_3_	m	0.5–0.7	C2
	LDL1/VLDL1	0.74–0.85	CH_3_(CH_2_)_n_/ CH_3_CH_2_CH_2_C=	m	>0.7	C2
	LDL2/VLDL2	1.16–1.25	(CH_2_)_n_/CH_2_CH_2_CH_2_CO	m	>0.7	C2
	lipids mainly VLDL	1.88	CH=CHCH_2_	m	0.5–0.7	C2
	lipids	2.24	CH_2_CO	m	>0.7	C2
	lipids	3.17	C=CCH_2_C=C	m	>0.7	C2
	polyunsaturated fatty acids	5.34–5.44	CH=CHCH_2_CH=CH, =CHCH_2_CH_2_	m	>0.7	C2

For urine samples, the clear separation along the first component (Supplementary Fig. S5a), based on the corresponding S-line plot (Supplementary Fig. S5b), was attributed to the metabolites presented in Table 4.

For maternal serum, the extracted OPLS-DA model (Supplementary Fig. S5c) clearly discriminated the samples along the first component and indicated that the samples belonging to C2 were characterized by higher levels of lipoproteins, as depicted in the corresponding S-line plot (Supplementary Fig. S5d). These lipoproteins are presented in Table 4.

Finally, the use of validation steps (P < 0.05, Permutation testing, and ROC curves) confirmed that the results of all OPLS-DA models for each substrate were unbiased and reliable as described in the Supplementary Figs S6, S7, and S8.

### Metabolite pathway analysis

After feature selection, metabolites exhibiting AUROC > 0.7 in AF samples were subjected to pathway analysis in order to relate the framed metabolic patterns to the most relevant pathways. The result of the pathway analysis for AF samples is depicted in Supplementary Fig. S9. Specifically, the pathways of importance containing at least 2 compounds involve the aminoacyl-tRNA and the citric acid cycle.

Metabolite Set Enrichment Analysis (MSEA), using Metaboanalyst 3.0^42^, was performed for the metabolites in AF exhibiting AUROC > 0.7. MSEA monitors whether these metabolites are represented more often than expected by chance and in an attempt to identify biologically meaningful patterns. The results pointed to protein biosynthesis as the only statistically significant pathway (P < 0.05) (Supplementary Fig. S10).

## Discussion

The present study is the first report attempting to probe the effects of maternal habitual diet on human AF composition, suggesting that the nutritional environment of AF is sensitive to female diet in the 2^nd^ trimester of pregnancy. The metabolic modifications in AF induced by different maternal dietary habits could be linked to amino acid metabolism, glucose metabolism, and citric acid cycle.

A detailed comparative analysis of our results against published literature is not feasible due to the limited data available in this area. To the best of our knowledge, there is only one relevant study in humans by Felig *et al*.^20^, where paralleling changes were reported in maternal plasma and AF, i.e. increase in branched-chain amino acids and decrease in alanine levels, after 84–90 hours of fasting, at 16–22 weeks of gestation. Evidence from animal models indicates, also, that maternal diet can affect the complex nutrient matrix of AF^14–19^.

**Figure 5:**
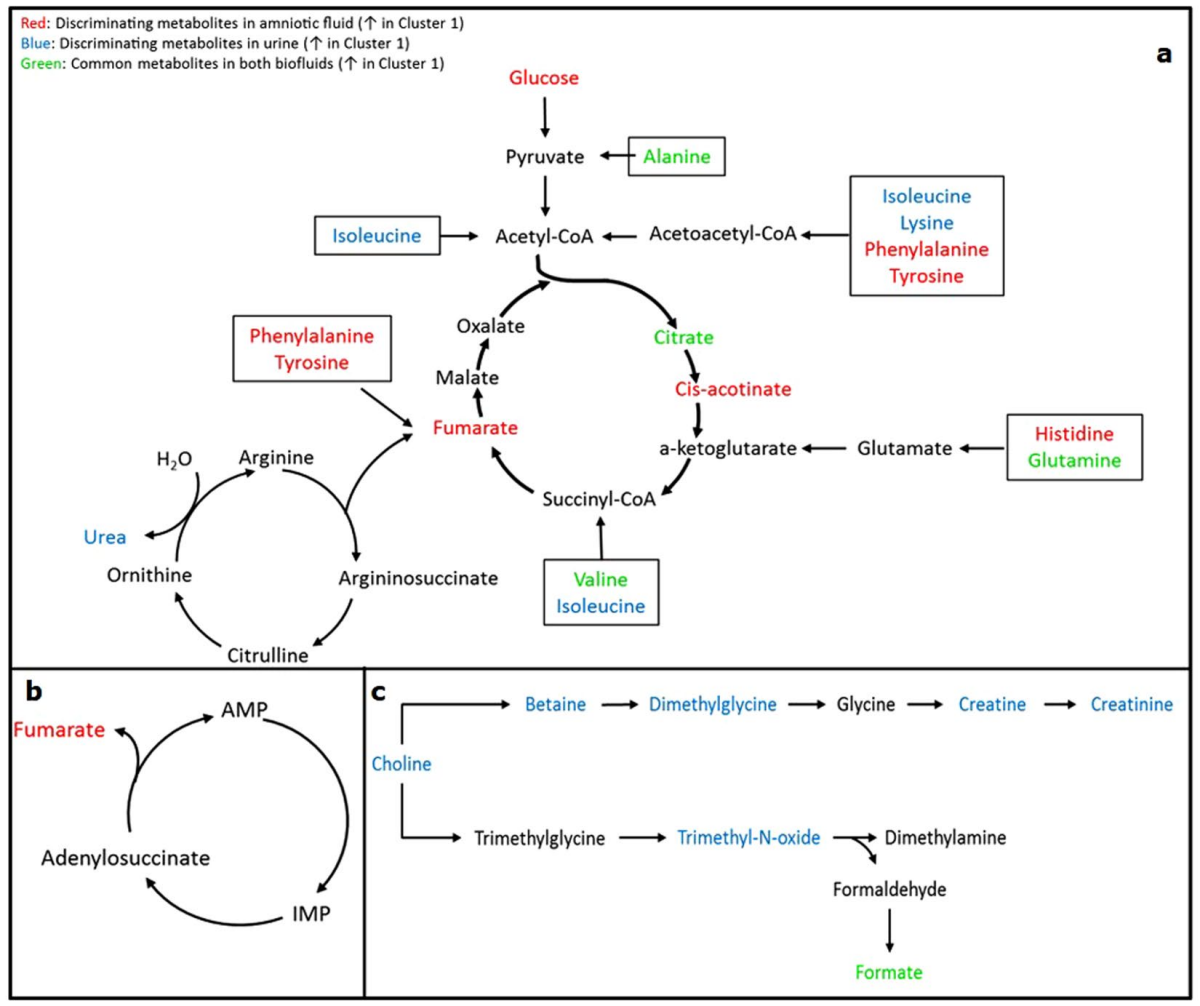
Schematic diagram illustrating the metabolic pathways that are possibly influenced by maternal habitual diet: (**a**) energy metabolism, amino acids metabolism, and urea cycle; (**b**) fumarate generation during purine biosynthesis; (**c**) choline metabolism.

To facilitate the interpretation of our results, perturbed metabolites identified in AF, as well as their associated metabolic pathways, are depicted in Fig. 5. As shown in Fig. 5a, higher AF glucose levels were recorded for C1, although no difference in maternal serum glucose was found between the two dietary clusters. Koski and Fergusson^14^ reported – in rats being in a post-absorptive (fed) state – no significant changes in maternal blood glucose concentrations, but increases in AF glucose with increases in maternal dietary carbohydrate intake levels. Considering that there was no statistically significant difference between C1 and C2 either for the time since last meal, or for carbohydrate intake, a plausible explanation for our finding may be related to the quality of carbohydrate. The latter can be linked to the higher dietary GI of C1 which may, in turn, alter the rate of glucose flux. It is important to note that glucose is the major energy substrate for fetal development and may be utilized, through conversion to other compounds, for protein synthesis and new tissue growth^43^. Commensurate with the higher AF glucose in C1, the higher levels of the essential amino acids histidine, phenylalanine, valine, and of the non-essential ones, alanine and tyrosine (Fig. 5a), may simply indicate either a differential rate to meet the requirements for elementary building blocks or a comparative under-use in gluconeogenesis. Furthermore, the increased levels of valine in AF of C1 may contribute to the balance between the branched chain amino acids^44^, known to be the major source of nitrogen for the ureogenic amino acids, alanine and glutamine^45^. At this point it is tempting to hypothesize that the relative increases in AF metabolites of C1 might reflect an increased energy availability ensuing from the increased fluxes of substrates, as echoed by the different combination of dietary factors characterizing this cluster. This is further supported by the fact that citrate is also elevated in C1, exhibiting a similar trend with glucose; a finding that is in agreement with the results found by Wan *et al*.^19^, who reported that citrate fluctuation in AF corresponds to glucose level fluctuations. The relative abundance of citrate in tandem with *cis*-acotinate in C1 may, thus, also suggest a differential management of the metabolic pool, since citric acid cycle may, also, provide building blocks for important biomolecules (Fig. 5a). Whether these changes direct/promote a metabolic switch that affects fetal development/growth, as well as the risk to develop chronic diseases in adult life, remains an open question. Accordingly, the relative higher AF levels of fumarate, observed in C1, could be related to a distinct intermediary metabolic rate, given that fumarate is situated at an important metabolic junction, performing key physiological functions; i.e. (i) its synthesis links the urea and the citric acid cycles (Fig. 5a); (ii) fumarate is involved in the cataplerotic pathway of phenylalanine and tyrosine (Fig. 5a); (iii) fumarate is generated during purine biosynthesis (Fig. 5b), where formate – increased in AF of C1, as well – acts as a potential alternative single-carbon source. We dare to speculate that formate in AF may be a marker of the biological consequences of the quality of dietary intake, since it is suggested in the literature^46^ that formate is excreted as a secondary metabolite in the case of high GI diets. The above speculation is further supported by the fact that, in the last decade, important evidence has shown that during pregnancy, maternal gut microbiota or its metabolic products may be transferred to the fetus through the placenta^47,48^.

With respect to pregnancy and fetal nutrition, it was of interest to explore how the habitual dietary patterns would be reflected in the metabolomic data of maternal compartments, i.e. urine and serum. As expected, ^1^H NMR spectra of maternal urine allowed the identification of metabolites associated with the two dietary patterns. Urine is the biofluid most frequently used to study nutrient intake^49–51^, since it is the body’s liquid waste repository^21^. At this point it is worth mentioning that during pregnancy the urine metabolome is also influenced by the remarkable physiological forces set in motion by conception^52^. Within this frame, the excretion of alanine increases rapidly in early pregnancy and continues to increase as pregnancy proceeds^53^. However, since the two clusters did not differ in gestational age, the increased alanine excretion in C1 could be attributed to dietary intake. Holmes *et al*.^54^ reported that urinary excretion of alanine is higher in people consuming a predominantly animal diet, proposing a direct association between excreted alanine and blood pressure. Furthermore, Bertram *et al*.^55^ and Dragsted^56^ ascribed the higher levels of excreted urea to the higher red meat consumption and higher animal protein intake. In line with our observation, in the study conducted by O’Sullivan *et al*.^57^, higher urinary dimethylglycine and trimethylamine N-oxide (TMAO) were identified in a dietary cluster characterized by higher habitual intakes of white bread, sugars/preserves, red meat, red-meat dishes, and meat products, and a lower contribution from vegetables. Interestingly, dimethylglycine, TMAO, creatine, and creatinine, as well as choline, betaine, and formate – increased in C1 - are metabolically linked in two different pathways of “choline metabolism” (Fig. 5c); (i) choline oxidation into betaine and (ii) bacterial degradation of choline into TMAO by the gut microbiome. Regarding formate excretion, it has been reported to be elevated in a group of adults following a high GI diet^46^. Moreover, the presence of bile acids in maternal urine during pregnancy has been suggested in the literature^31,38^; however, the higher levels of these important signalling biomolecules in C1 merit further investigation, preferably by quantitative LC-MS/MS analysis.

Regarding the maternal circulatory metabolome, it was dominated by signals from lipids and lipoproteins. Hyperlipidemia of normal pregnancy results in high blood HDL, LDL, VLDL, and triglycerides, accompanied by increases in the length of fatty acid chain and the degree of unsaturation^38,58–60^. The present study showed that this expected increase in maternal lipids was, further, promoted in women of C2, whose dietary preferences were associated with higher total lipid, monounsaturated and polyunsaturated fatty acids intakes, compared to C1. It is of interest to mention that in a very recent study^61^, higher blood total cholesterol levels were recorded in pregnant women following a dietary pattern characterized, among others, by higher intakes of fruits, vegetables, whole grains, and low-fat dairy. However, due to the strong influence of pre-pregnancy lipid levels and maternal hormonal status during pregnancy on lipid metabolism^59,62^, no clear biochemical interpretation may be advanced at least at this stage.

Our results express the potential prospects of using metabolomics in the quest for habitual diet induced metabolic signals in AF, in spite of existing limitations related to genetic background information. Furthermore, to obtain a more accurate picture of the overall metabolic changes, confounding factors, such as maternal hormonal status have to be assessed. Nevertheless, the results of the current study have to be interpreted in the light of its strengths, concerning the experimental approaches undertaken. Firstly, the fact that in the present study we analysed AF after excluding samples from pregnancies that (i) were complicated by structural malformations and/or chromosomal abnormalities of the fetus, (ii) were characterised by obstetrical or medical disorders, or (iii) ended in delivering a small or large for gestational age infant, eliminated the potential overlapping with metabolic effects attributable to these aforementioned fetal/maternal disturbances^12,24–27,29,30,33–35,40^. Moreover, the parallel analyses of the three biological specimens (obtained at the time of genetic amniocentesis) provide complementary information of fetal metabolism, through AF analysis, and maternal metabolism, by the excretive and circulating characteristics of the mother. The great advantage of using untargeted metabolomics is that all metabolites (those present in detectable concentrations) are measured simultaneously. Thus, metabolic profiling of AF, as well as of maternal urine and serum, in conjunction with detailed recording of the maternal complex dietary preference background, does offer a more holistic approach that leads to a better description of the metabolic trajectory of the fetus, with respect to maternal nutrition.

In conclusion, our data provide the first evidence to suggest that maternal habitual dietary patterns influence the metabolic profile of human AF. Notably, very recently, Kermack *et al*.^63^ reported that differences in women’s diet quality can alter the amino acid concentration of human uterine fluid. Taken together, these results highlight the need to raise nutritional awareness and provide a framework for further research on the effect of maternal nutrition on pregnancy evolution and outcome, using a combination of biological matrices and analytical platforms.

## Methods

### Study population

The present study was part of the Embryometabolomics project^64^. Women in the second trimester of pregnancy were invited to participate in the Embryometabolomics project, while visiting the 1^st^ Department of Obstetrics and Gynecology, Papageorgiou General Hospital, Thessaloniki, Greece, to undergo amniocentesis for prenatal diagnosis. Indications for amniocentesis included maternal age, ultrasound markers, family history of genetic disorders, previous fetal aneuploidy, and maternal anxiety. Women were informed about the objectives of the Embryometabolomics project and gave their signed consents; women who agreed to participate completed a structured interview concerning maternal demographic/anthropometric characteristics, while respective samples of AF were stored at −80 °C until further analysis.

The methodological strategy of the present study is depicted in Fig. 1. From those women who were enrolled in the Embryometabolomics project, dietary information was available from 72 women (Fig. 1) and, as such, they were recruited for the present study. Finally, 65 were included, as they met the following criteria: (a) singleton pregnancy, (b) absence of structural malformations and/or chromosomal abnormalities of the fetus, (c) delivery of an appropriate for gestational age infant (birth weight between the 10^th^ and 90^th^ centile), (d) absence of obstetrical or medical complications, such as preeclampsia or gestational diabetes mellitus, and (e) dietary energy intake within the allowable range for pregnant women^65,66^.

Ethical approval was obtained from the Bioethics Committee of the Medical School of the Aristotle University in Thessaloniki, Greece (A19479–26/2/08). All methods were performed in accordance with the relevant guidelines and regulations.

### Biofluid collection

All biological specimens were collected under non-fasting conditions, due to medical restrictions in controlling/limiting pregnant women’s diet. AF specimens were retrieved using a 20 G spinal needle under ultrasound guidance. Blood samples were collected, allowed to clot, and centrifuged at 3500 g for 5 min; serum was, then, aliquoted. Spot urine samples were collected in sterile containers. Biofluids were stored at −80 °C until further preparation and analysis.

### Dietary assessment

Dietary assessment was carried out using a semi-quantitative Food Frequency Questionnaire (FFQ) validated for pregnant women^41^. All dietary information were collected prior the antenatal appointment via personal interview by a registered dietician or a well-trained interviewer (food scientist-nutritionist). For the conversion of women responses into dietary data, the Microsoft excel database was used as described by Athanasiadou *et al*.^41^.

### Statistical Analyses for identification of dietary patterns

HCA^67,68^ was used to identify groups of women consuming a similar dietary pattern. Prior to cluster analysis, the individual food items were categorized into 20 predefined food groups – as shown in Table 1 – based on similarities in their nutrient profiles and culinary usage/parameters with potential relevance to food culture^69–79^.

For entry into the cluster analysis, the percentage of energy contributed by each of the 20 food groups was selected as input variable. Cluster construction was based on Ward’s minimum variance criterion^80^, while the squared Euclidian distance was used as a dissimilarity measure^67^. The food-group data were transformed into standardized z scores, before clustering, so that they had equal weights when distances were computed^72^. The theoretical background for adopting the above mentioned methodological scheme for HCA is reported by Taxidis *et al*.^81^.

Runs of cluster formation were performed to establish the best cluster configuration. Criteria for cluster solutions were nutritional meaningfulness and a reasonable sample size. The solution was confirmed by the tree diagram resulting from the Ward method of cluster analysis. Furthermore, Discriminant Analysis was carried out to examine the classification ability of the cluster solution^82^. The statistical significance of the final cluster solution was evaluated with the upper-tailed rule, using the Clustan ver. 5.27^83^.

In order to compare normally and non-normally distributed parameters between the clusters, Student’s *t* test for independent samples and Mann-Whitney test were used, respectively. In Mann-Whitney test, the observed significance level (P-value) was computed with the Monte-Carlo simulation method^84^ utilizing 10000 random samples. All statistical analyses were performed with SPSS v.15.0 (SPSS Inc., Chicago, IL). The significance level was predetermined at P  < 0.05.

### NMR spectroscopy

#### Sample preparation

All NMR spectra were acquired on a Varian-600MHz NMR spectrometer equipped with a triple resonance probe {HCN} at 25 °C. The Carr-Purcell-Meiboom-Gill (CPMG) pulse sequence was applied with 128 transients collected with 64 K data points to AF, urine, and serum samples. The samples were thawed at room temperature 60 min before performing the NMR experiments.

*AF*: 400 μL D_2_O and 150 μL phosphate buffer in D_2_O were added in lyophilized samples. After centrifugation (4500 g, 15 °C, 5 min), 50 μL sodium maleate was added as internal standard to 500 μL of the supernatant and the sample was transferred to 5 mm NMR tubes.

*Urine*: Samples were prepared by adding 150 μL phosphate buffer in D_2_O to 400 μL urine. After centrifugation (10000 g, 4 °C, 10 min), 50 μL sodium trimethylsilyl propionate (TSP) was added as internal standard to 500 μL of the supernatant and transferred to 5 mm NMR tubes.

*Serum*: Samples were prepared by adding 140 μL phosphate buffer in D_2_O to 400 μL serum. After centrifugation (10000 g, 4 °C, 10 min), 50 μL sodium maleate was added as internal standard to 500 μL of the supernatant and transferred to 5 mm NMR tubes.

Sodium maleate was chosen as reference standard for serum and AF since it is suitable for CPMG pulse sequence and provides a distinct peak in the ^1^H NMR spectrum^85^. Relaxation delay was set to 6 s. Proton spectra were referenced at the resonance peak of sodium maleate (5.95 ppm). Receiver Gain was kept constant for all acquisitions.

A series of 2D experiments, gCOSY, zTOCSY, gHMBCad, gHSQCad were recorded at 25 °C and permitted the assignment of metabolites. The acquisition parameters for 2D NMR experiments are described in the Supplementary Material. The interpretation of 2D spectra was performed with the use of MestReNova v.10.1 software. The identification procedure was also assisted by literature data^12,24,28,31,38^, a reference metabolite ^1^H NMR database (Chenomx NMR Suite 7.0) and an in-house fully automated metabolite identification platform^86^.

All ^1^H NMR spectra were phase and baseline corrected.

#### Data reduction and spectral alignment

The ^1^H NMR spectra were reduced into buckets of 0.0001 ppm and the D_2_O (4.6–4.8 ppm) region was removed. The spectra were aligned, normalized to the standardized area of the reference compound and converted to ASCII format using the Mnova processing template.

### Statistical Analyses for ^1^H NMR data

The SIMCA-P version 14.0 (Umetrics, Umeå, Sweden) was facilitated. The spectral data were mean-centered Pareto scaled (Par) and the PCA, as well as the OPLS-DA models were extracted at a confidence level of 95%. The mathematical background and applications of these methods have been extensively discussed elsewhere^87^.

The online software Metaboanalyst 3.0 was utilized^42^ for biomarker discovery, classification and pathway mapping. A hypergeometric test using over-representation analysis and pathway topology analysis related these metabolites to metabolic pathways.

#### Identification of important Features in the OPLS-DA models

Feature selection for the OPLS-DA models was based on variable importance in projection (VIP) scores larger than 0.7 and P(corr) > 0.2 to reveal the variables which bear class discriminating power. S-line plots were facilitated to pinpoint those metabolites that contribute to the samples’ discrimination.

### Model Validation

The validation steps followed by Fotakis *et al*.^85^ were implemented in this work, as described in the Supplementary Material.

### Data availability

All data generated or analyzed during this study are included in this published article (and its Supplementary Information files).

## Electronic supplementary material

Supplementary Material
