# Supplementary material for: 1H NMR-based metabolomics reveals the effect of maternal habitual dietary patterns on human amniotic fluid profile
Source: Sci Rep. 2018 Mar 6;8:4076. doi: 10.1038/s41598-018-22230-y (PMC5840288; doi:10.1038/s41598-018-22230-y)
Supplement: Supplementary file 1 — Supplementary Material [file 41598_2018_22230_MOESM1_ESM.pdf]

^{1}H NMR-based metabolomics reveals the effect of maternal habitual dietary patterns on human amniotic fluid profile

Maria Fotiou[1,#], Charalambos Fotakis[2,#], Foteini Tsakoumaki[1], Elpiniki Athanasiadou[3], Charikleia Kyrkou[1], Aristea Dimitropoulou[1], Thalia Tsiaka[2], Anastasia Chrysovalantou Chatziioannou[4], Kosmas Sarafidis[5], George Menexes[6], Georgios Theodoridis[4], Costas G. Biliaderis[1], Panagiotis Zoumpoulakis[2,*], Apostolos P. Athanasiadis[7,*] & Alexandra-Maria Michaelidou[1,*]

[1]Department of Food Science and Technology, School of Agriculture, Aristotle University of Thessaloniki, Greece.

[2]Institute of Biology, Medicinal Chemistry and Biotechnology, National Hellenic Research Foundation, Athens, Greece.

[3]1st Department of Obstetrics and Gynecology, School of Medicine, Aristotle University of Thessaloniki, Greece.

[4]School of Chemistry, Aristotle University of Thessaloniki, Greece.

[5]1st Department of Neonatology, School of Medicine, Aristotle University of Thessaloniki, Greece.

[6]Department of Field Crops and Ecology, School of Agriculture, Aristotle University of Thessaloniki, Greece.

[7]3rd Department of Obstetrics and Gynecology, School of Medicine, Aristotle University of Thessaloniki, Greece.

*These authors contributed equally to this work.

Correspondence and requests for materials should be addressed to P.Z. (NMR metabonomics; email: pzoump@eie.gr), A.P.A. (clinical data; email: apostolos3435@gmail.com), or A-M.M. (nutritional assessment; email: amichail@agro.auth.gr)

Figure S1. ^{1}H NMR spectra of a urine sample with annotation on the identified metabolites. (a) 1:lactate; 2:alanine; 3:3-amino-isobutyrate; 4:valine; 5:isoleucine; 6:leucine; 7:isobutyrate; 8:methyl-succinate; 9:L-fucose; 10:lysine; 11:bile acids (2-hydroxybutyrate); 12:3-methyl-2-oxovalerate; 13:3-hydroxyisovalerate; 14:2-hydroxyisobutyrate. **(b)** 15:β-alanine; 16:2-hydroxyglutarate; 17:lysine; 18:citrate; 19:acetate; 20:N-acetylaspartate; 21:N-acetylneuraminate; 22:L-glutamine; 23:acetone; 24:aminoadipate; 25:acetoacetate; 26:pyroglutarate; 27:pyruvate; 28:isobutyrate; 29:dimethylamine; 30:methylguanidine; 31:creatine; 32:creatinine; 33:trimethylamine; 34:dimethylglycine; 35: isocitrate. **(c)** 36:urea; 37:D-glucose; 38:sucrose; 39:cis-acotinate; 32:creatinine; 31:creatine; 1:lactate; 40:L-cystine; 41:gluconate; 42:glycolate; 43:serine; 44:hippurate; 45:trigonelline; 46:threonine; 47:choline; 48:trimethylamine-N-oxide; 49:betaine; 50:taurine; 51:1-methylnicotinamide; 52:guanidoacetate; 53:glycine. **(d)** 44:hippurate; 45:trigonelline; 54:formate; 55:τ-methylhistidine; 56:1-methylnicotinamide 57:N-phenylacetylglycine; 58:phenol; 59:tyrosine; 60:histidine; 61:π-methylhistidine; 62:γ-glutamylphenylalanine; 63:imidazole; 64:mandelate; 65:indoxylsulfate.

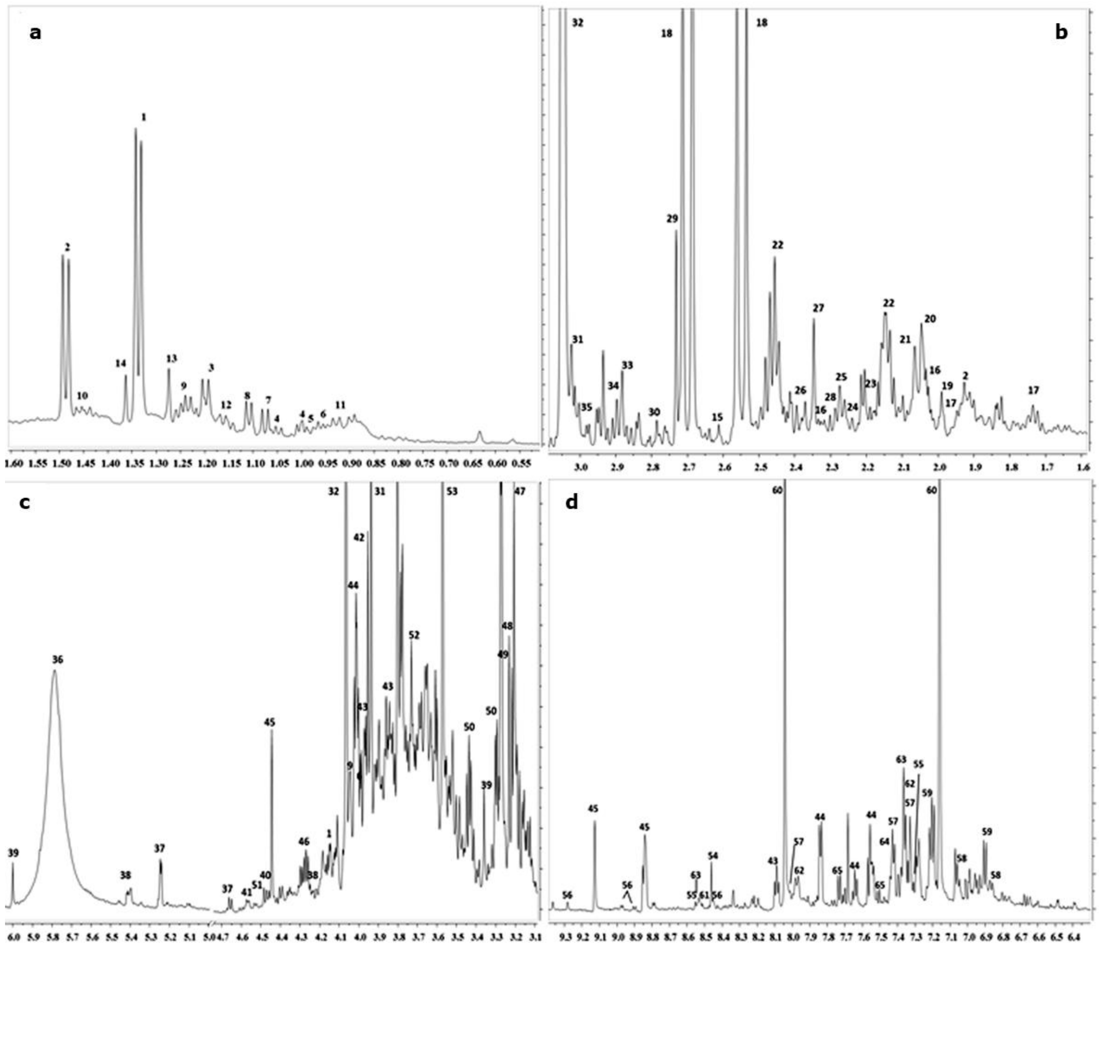

Figure S2. ^{1}H NMR spectra of a serum sample with annotation on the identified metabolites. 1:valine; 2:leucine; 3:isoleucine; 4:cholesterol-VLDL; 5:LDL2/VLDL2; 6:lactate; 7:alanine; 8:lysine; 9:arginine; 10:acetate; 11: *n*-acetylated glycoprotein 1; 12: *n*-acetylated glycoprotein 2; 13:n-acetyl glutamine; 14:glutamine; 15:glutamate; 16:citrate; 17:acetone; 18:acetoacetate; 19:creatine; 20:creatinine; 21:asparagine; 22:α-D-glucose; 23:β-D-glucose; 24:methylamine; 25:glycerol; 26:glycine; 27:histidine; 28:tyrosine; 29:unsaturated lipid; 30:choline; 31:betaine; 32:phosphocholine; 33:threonine; 34:formate; 35:phenylalanine.

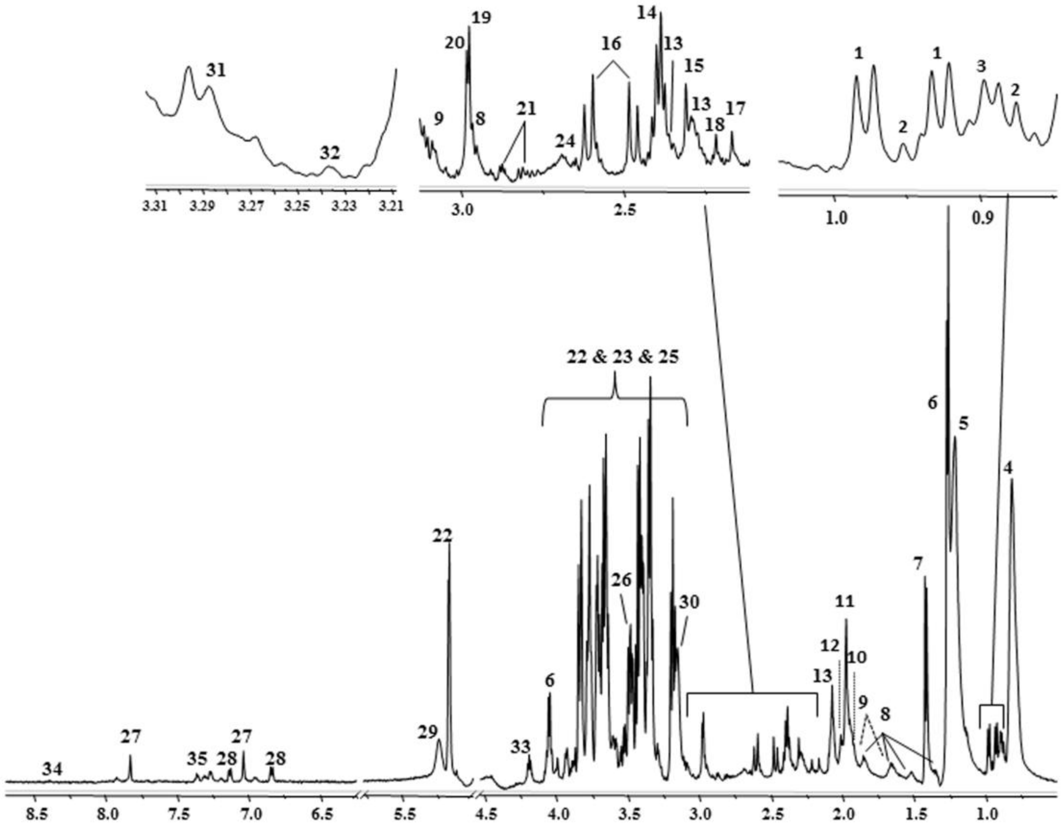

Figure S3. (a) PCA model of urine samples. A=5; N=36; R^2(cum)=0.73; Q^2(cum)=0.56. **(b) PCA model of serum samples**. A=2; N=56; R^2(cum)=0.62; Q^2(cum)=0.58. ● Cluster 1 (C1) ● Cluster 2 (C2).

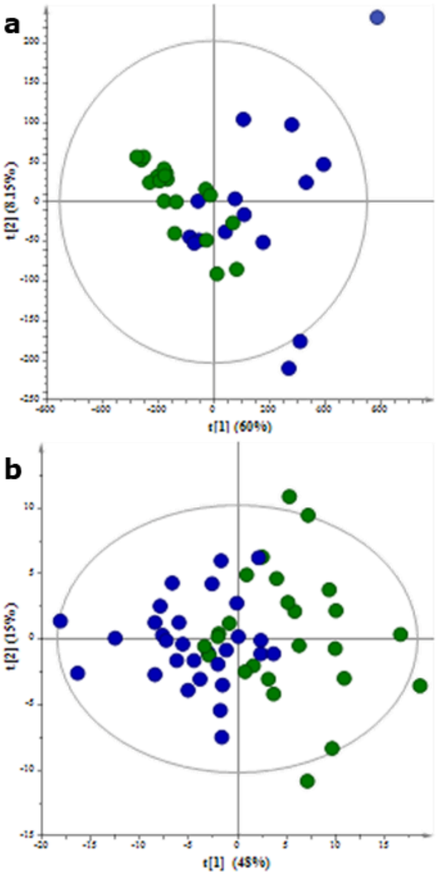

Figure S4. ROC curves (AUROC>0.7) and box plots of metabolites contributing to the discrimination of amniotic fluid samples.

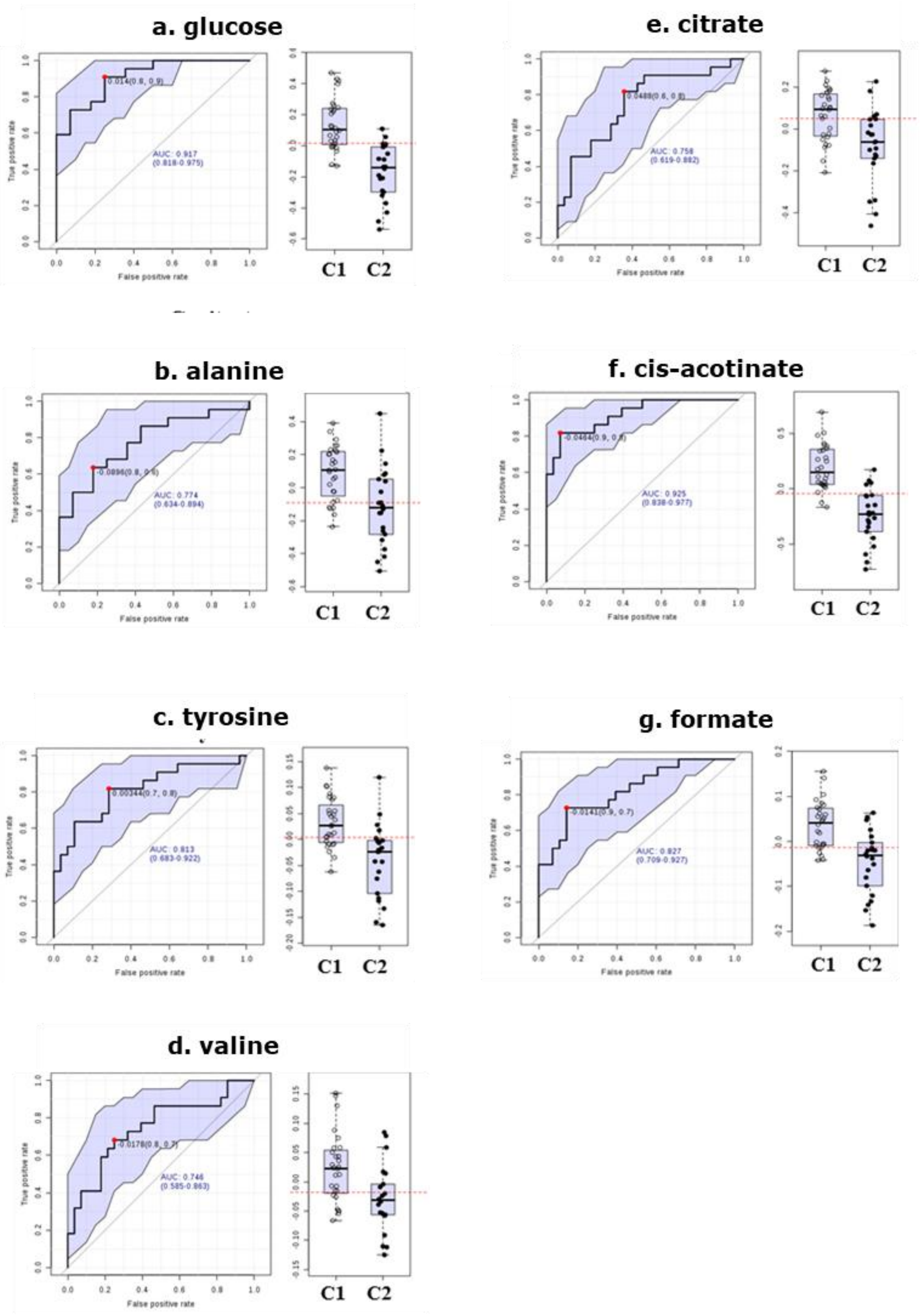

Figure S5. (a) OPLS-DA model of urine samples. A=1+1; N=31; R^2X(cum)= 0.69; R^2Y(cum)=0.77; Q^2(cum)=0.60. **(b) S-Line plot** (1:bile acids; 2:valine; 3:isoleucine; 4:leucine; 5:isobutyrate; 6:methyl succinate; 7:3-hydroxyisovalerate; 8:2-hydroxyisobutyrate; 9:lysine; 10:alanine; 11:2-hydroxyglutarate; 12:L-glutamine; 13:pyroglutarate; 14:citrate; 15:creatine; 16:creatinine; 17:dimethylglycine; 18:choline; 19:trimethylamine-N-oxide; 20:betaine; 21:urea; 22:imidazole, higher in Cluster 1). **(c) OPLS-DA model of serum samples**. A=1+1; N=50; R^2X(cum)=0.61; R^2Y(cum)=0.67; Q^2(cum)=0.54. **(d) S-Line plot** (1:cholesterol-VLDL C_{18}-CH_3; 2:LDL1/VLDL1 $CH_3(CH_2)n$/ $CH_3CH_2CH_2C$=; 3:LDL2/VLDL2 $(CH_2)n/CH_2CH_2CH_2CO$; 4:lipids mainly VLDL; 5:lipids CH_2CO; 6:lipids $C=CCH_2C=C$; 7:poly-unsaturated fatty acids $CH=CHCH_2CH=CH$, $=CHCH_2CH_2$, higher in Cluster 2). ● Cluster 1 (C1) ● Cluster 2 (C2).

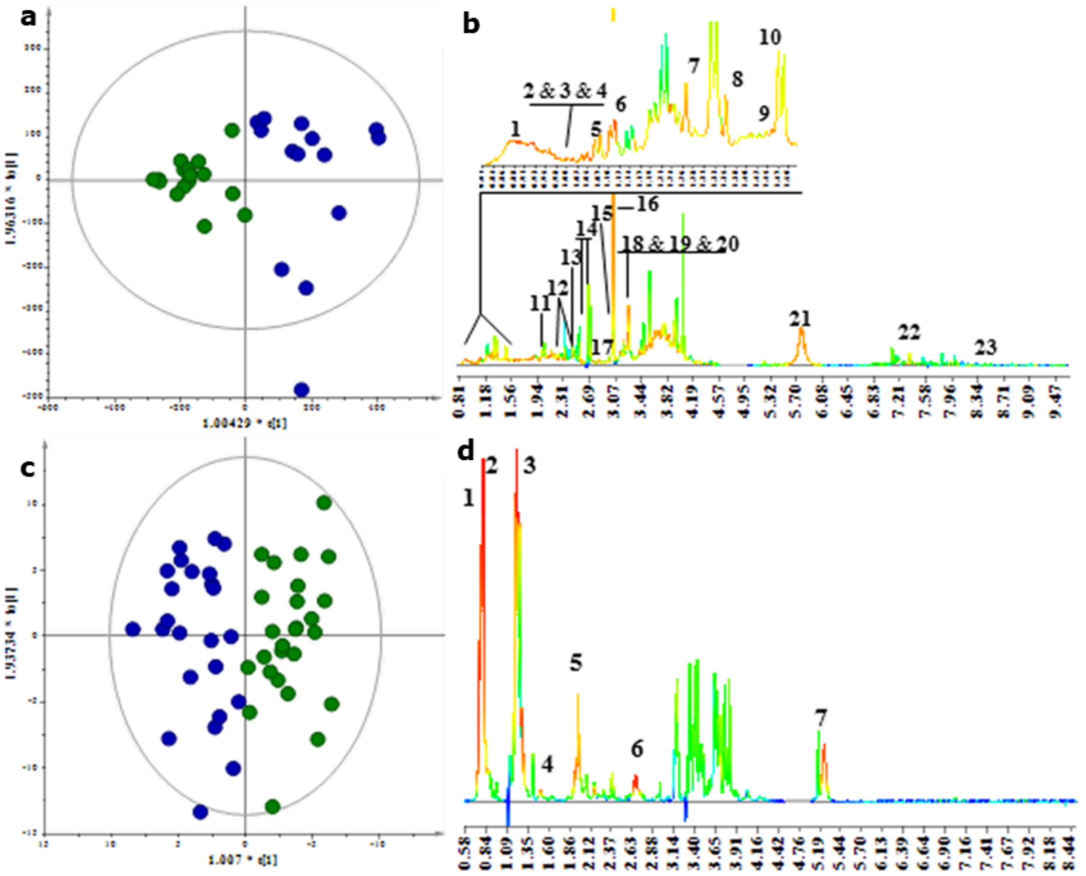

Figure S6. (a) ROC curve and (b) permutation testing for the OPLS-DA model of the amniotic fluid samples.

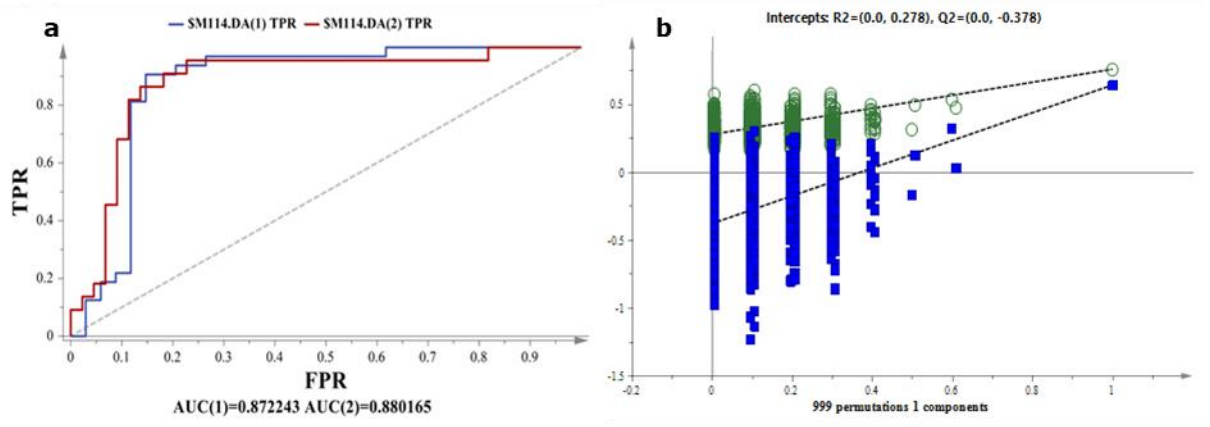

Figure S7. (a) ROC curve and (b) permutation testing for the OPLS-DA model of the urine samples.

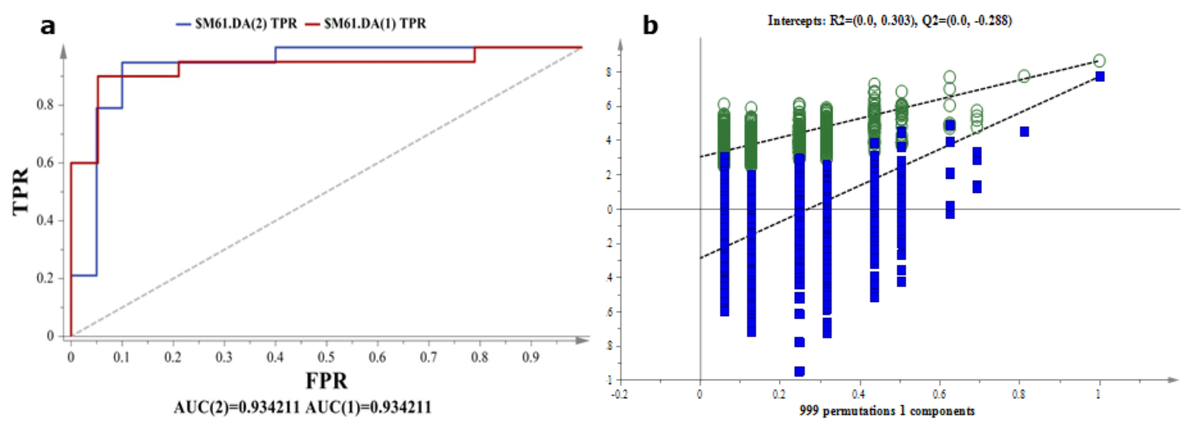

Figure S8. (a) ROC curve and (b) permutation testing for the OPLS-DA model of the serum samples.

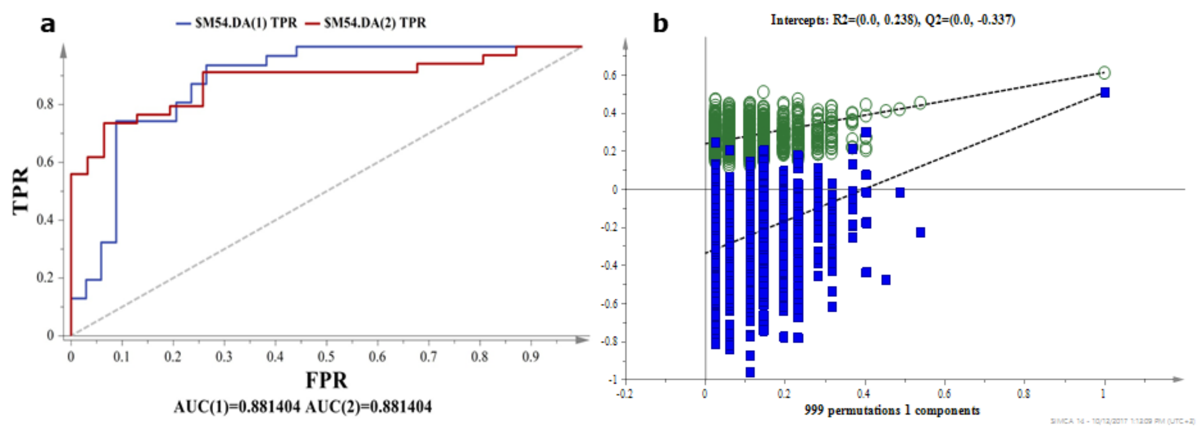

Figure S9. (a) Summary and (b) results of pathway analysis on amniotic fluid samples (in bold, pathways of importance)**.**

a

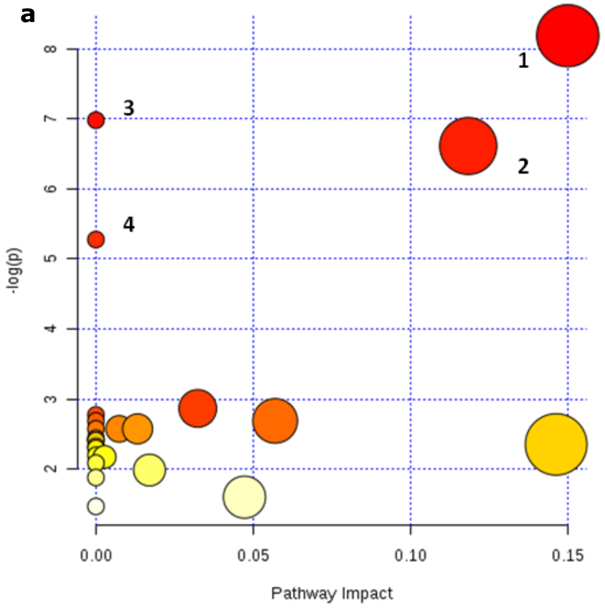

b

	Pathway	Total	Expected	Hits	Raw p	-log (p)	Holm adjust	FDR	Impact
1	Glyoxylate and dicarboxylate metabolism	50	0.145410	3	0.000279	8.185800	0.022287	0.022287	0.150020
**2**	**Aminoacyl-tRNA biosynthesis**	**75**	**0.218110**	**3**	**0.000930**	**6.980500**	**0.073456**	**0.035838**	**0.000000**
**3**	**Citric acid cycle**	**20**	**0.058164**	**2**	**0.001344**	**6.612200**	**0.104830**	**0.035838**	**0.118380**
4	Nitrogen metabolism	39	0.113420	2	0.005105	5.277600	0.393050	0.102090	0.000000
	Valine, leucine and isoleucine degradation	40	0.116330	1	0.110820	2.199900	1.000000	0.477670	0.000000

Figure S10. (a) Correlation network based on the amniotic fluid samples and (b) results of the Metabolite Set Enrichment Analysis.

a

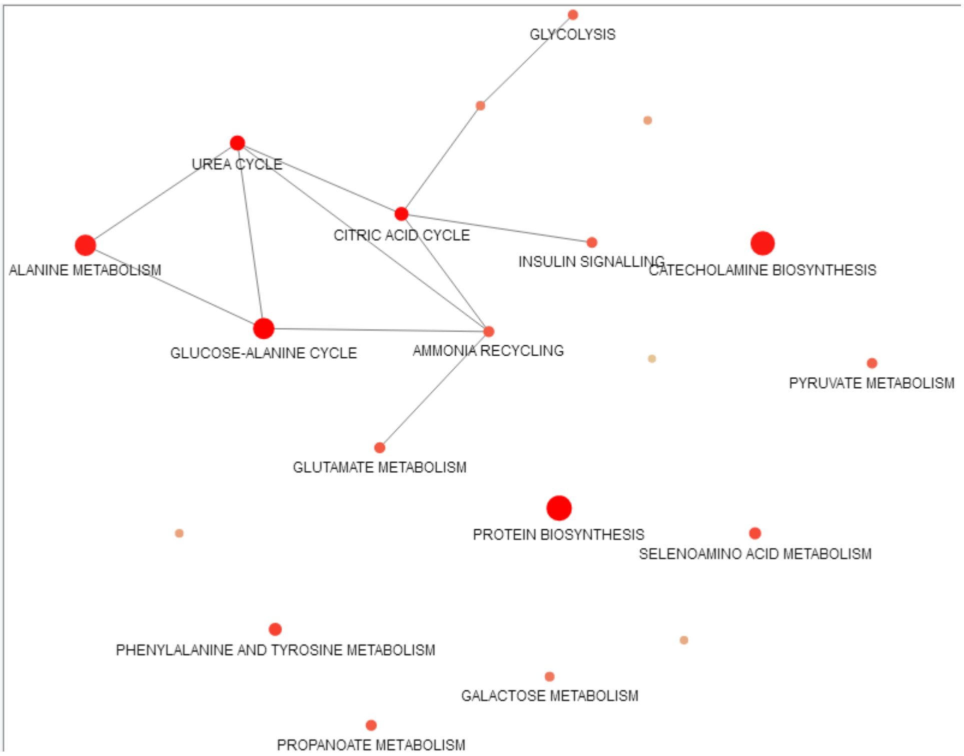

b

Metabolite Set	Total	Hits	Expect	P value	Holm P	FDR
Protein biosynthesis	19	4	0.184	1.34E-05	0.00107	0.00107
Glucose-alanine cycle	12	2	0.117	0.00519	0.41	0.208
Urea cycle	20	2	0.194	0.0144	1	0.383
Catecholamine biosynthesis	5	1	0.0485	0.0477	1	0.912
Alanine metabolism	6	1	0.0583	0.057	1	0.912
Phenylalanine and tyrosine metabolism	13	1	0.126	0.12	1	1
Selenoamino acid metabolism	15	1	0.146	0.137	1	1
Ammonia recycling	18	1	0.175	0.163	1	1
Propanoate metabolism	18	1	0.175	0.163	1	1
Glutamate metabolism	18	1	0.175	0.163	1	1
Insulin signaling	19	1	0.184	0.171	1	1
Pyruvate metabolism	20	1	0.194	0.179	1	1
Glycolysis	21	1	0.204	0.187	1	1
Citric acid cycle	23	1	0.223	0.203	1	1
Purine metabolism	45	1	0.437	0.363	1	1

Acquisition parameters for 2D NMR experiments

The acquisition parameters for gCOSY were: spectral width (SW) 7225.4 Hz, t1 increments 256, acquisition time 0.150 s, number of scans 128, 1084 data points, receiver gain 30 and relaxation delay 1 s. The acquisition parameters for zTOCSY was performed with spectral width (SW) 7225.4 Hz, t1 increments 256, number of scans 128, acquisition time 0.283 s, 2048 data points, receiver gain 30 and relaxation delay 1 s. The acquisition parameters for gHSQCad were: f2 spectral width (SW) 7225.4 Hz, f1 spectral width (SW) 30165.9 Hz, t1 increments 256, number of scans 128, acquisition time 0.150 s, 1084 data points, receiver gain 30 and relaxation delay 1 s. The acquisition parameters for gHMBCad were: f2 spectral width (SW) 7225.4 Hz, f1 spectral width (SW) 36199.1 Hz, t1 increments 256, number of scans 128, acquisition time 0.150 s, 1084 data points, receiver gain 40 and relaxation delay 1 s.

Model Validation - Validation steps

The quality of models (PCA/OPLS-DA) was described by the goodness-of-fit R^2 ($0 \leq R^2 \leq 1$) and the predictive ability Q^2 ($0 \leq Q^2 \leq 1$) values. The R^2 explains the variation, thus constituting a quantitative measure of how well the data of the training set was mathematically reproduced. The overall predictive ability of the model is assessed by the cumulative Q^2 representing the fraction of the variation of Y that can be predicted by the model, which was extracted according to the internal cross validation default method of SIMCA-P software.

The Q^2 is considered as de facto the default diagnostic parameter to validate PLS-DA models in metabolomics. In particular, all OPLS-DA models demonstrated high statistical values ($R^2 > 0.7$ and $Q^2 \geq 0.54$), the difference between the goodness-of-fit and the predictive ability remained always lower than 0.3 ($R^2X(cum) - Q^2(cum) < 0.3$) and the goodness of- fit never equaled to one ($R^2X(cum) \neq 1$). Therefore, since the extracted models abide by these rules, their robustness and predictive response are enhanced and over-fitting is effaced.

Regression models have been validated using cross validation analysis of variance (CV-ANOVA), with a P-value<0.05. CV scores plots were elicited in order to indicate the sensitivity of a model to the exclusion of an observation of the work set. Furthermore, permutation tests were employed (999 permutations) in order to evaluate whether the specific classification of two classes in a model are significantly better than any other models obtained by randomly permuting the original groups attribution. An additional measure of PLS-DA model validity included the extraction of receiver–operator characteristic (ROC) curves to assess the ability of the PLS latent variable Tpred to correctly classify the test set. The area under the ROC (AUROC) was calculated. A perfect discrimination corresponded to an AUROC equal to 1. Finally, classification lists, misclassification tables depicting the proportion of correctly classified observations in the prediction set, and most importantly external data set have been employed, further attesting to the generalizability of the models.
